# Sensor-Detected Differences in Behaviors of Older Drivers with Pre-MCI and Mild Cognitive Impairment vs. Unimpaired Drivers

**DOI:** 10.3390/s26010290

**Published:** 2026-01-02

**Authors:** Ruth M. Tappen, David Newman, Mónica Rosselli, Joshua Conniff, Subhosit Ray, Sonia Moshfeghi, Jinwoo Jang, KwangSoo Yang, Borko Furht

**Affiliations:** 1 Christine E. Lynn College of Nursing, Florida Atlantic University, 777 Glades Road, Boca Raton, FL 33431, USA; dnewma14@health.fau.edu (D.N.); rays2016@health.fau.edu (S.R.); 2 Department of Psychology, Charles E. Schmidt College of Science, Florida Atlantic University, 777 Glades Road, Boca Raton, FL 33431, USA; mrossell@fau.edu (M.R.); jconniff@fau.edu (J.C.); 3 Department of Civil, Environmental, and Geomatics Engineering, College of Engineering and Computer Science, Florida Atlantic University, 777 Glades Road, Boca Raton, FL 33431, USA; smoshfeghi2021@fau.edu (S.M.); jangj@fau.edu (J.J.); 4 Department of Electrical Engineering and Computer Science, College of Engineering and Computer Science, Florida Atlantic University, 777 Glades Road, Boca Raton, FL 33431, USA; yangk@fau.edu (K.Y.); bfurht@fau.edu (B.F.)

**Keywords:** Pre-MCI, telematics, accelerometer, older driver, driving behavior, mild cognitive impairment, naturalistic driving study, IVDR (In-Vehicle Data Recorder)

## Abstract

**Highlights:**

**What are the main findings?**

- Higher RPM, shorter average trips, and greater throttle variability were characteristic of drivers with Pre-MCI/MCI.
- More frequent hard braking, hard turns, higher mean speed, and lower average throttle (steadier pedal control) were characteristic of unimpaired drivers.

**What are the implications of the main findings?**

- The results support the hypothesis that driving behaviors of individuals with early (preclinical) impairment of cognition differ from those who are unimpaired.
- The differences observed are based upon the patterns of driving behaviors exhibited rather than in a single behavior.

**Abstract:**

*Background*: Research to identify changes in driving behavior that occur with the onset of Pre-MCI and MCI is an emerging area with many gaps still to be addressed. These gaps include limited use of objective, continuous measurement of driver behavior in real-life traffic conditions and comprehensive, biomarker-validated, cognitive evaluation based upon both testing and clinical ratings. Using these strategies, the questions addressed in this exploratory study are whether or not differences in driving behavior are indicative of Pre-MCI/MCI and which behaviors are most predictive of Pre-MCI/MCI. *Methods*: As part of a naturalistic longitudinal study, older drivers with a Montreal Cognitive Assessment score ≥ 19 had telematic sensors installed in their vehicles and underwent comprehensive cognitive assessment quarterly for three years. Thirty-six participants were classified as Unimpaired (n = 23) or Pre-MCI/MCI (n = 10/3) based upon a neuropsychological battery and diagnostic algorithm. A penalized generalized linear mixed-effects model (GLMM) with a logistic link and LASSO regularization was used to model Pre-MCI/MCI group membership vs. unimpaired as a function of ten trip-level telematic features (trip distance, hard acceleration, hard braking, hard turns, speed average, maximum speed, RPM average, fuel level, throttle average, and throttle variability) at the end of their first 12 months in the study. *Results*: Higher RPM, shorter average trips, and greater throttle variability predicted higher odds of Pre-MCI/MCI, while more frequent hard braking, hard turns, higher mean speed, and lower average throttle (steadier pedal control) predicted lower odds of Pre-MCI/MCI. *Conclusions*: The model clearly distinguished unimpaired older drivers from those with MCI or Pre-MCI, suggesting that distinct patterns of driver behavior may be related to levels of cognitive function.

## 1. Introduction

Safety, particularly reduction in crash risk, has long been a concern in research on older drivers. More recently, recognition that driving is a cognitively complex function has been combined with a heightened interest in early stages of cognitive decline, leading to the supposition that there may be identifiable changes in driving behavior that are reflective of the development of Pre-MCI and Mild Cognitive Impairment [1,2].

Research on the changes in driving behavior that reflect the onset of Pre-MCI and MCI is still in its infancy [3]. Roe 3] has cataloged the wide range of gaps in our knowledge of this topic that still exists. These include the association between driving behaviors in the early stages of the disease and changes in biomarkers, the potential confounding effects of sociodemographic variables, co-morbidities, the effects of medications, and the effects of the physical (e.g., weather, traffic) and social (e.g., the behavior of other drivers) environment in which the individual is driving. The physiological bases of these changes in driving behavior have not been fully explained, but it appears that both amyloid and tau-based pathology are associated with reduced driving capability, indicated by poor performance in on-road driving tests. Little is known as yet about the relationship of this decline in ability with changes identified in the newer blood-based biomarkers [3].

A number of different strategies have been used to measure driving behaviors and relate them to degrees of cognitive decline. These include the more traditional survey methodologies such as questionnaires, and standardized driving tests, as well as driving simulation. In-vehicle data recorders, and even smartphone technologies [4] are emerging as technologies that make these more naturalistic, continuous measures more accessible.

Of particular interest to the current study, the results of these studies have varied in terms of the factors identified as distinguishing cognitively unimpaired older drivers from those with mild cognitive impairment (MCI) or dementia. For example, a study of U.S. veterans who had been referred for an on-road driving evaluation found that 18% failed the on-road test, but the failure was not related to either age or clinical diagnosis [5]. Another study utilizing on-road driving tests found that age and lower scores on a digit symbol substitution test distinguished those with high numbers of unsafe driving behaviors during the test. The Driving Risk Checklist-25 [6,7] was quite successful in distinguishing those with MCI from those without MCI, yielding a specificity of 89.9% identifying those with MCI but a sensitivity of 45% in a sample of 387. In another sample of 185 older adults with MCI who were followed for three years, 144 were driving at baseline, but 27% stopped over the course of the 3-year study. Further, of the 50 participants who were diagnosed with dementia, all but one had stopped driving after receiving that diagnosis, suggesting that self-restriction may play an important role as dementia increases.

More specific to change in cognition, drivers with MCI have reported self-regulation related to driving at night and making turns across oncoming traffic, suggesting awareness of their limitations [8]. Drivers with dementia have reported reducing the amount of driving they do [9]. They have also reported lower driving-related ability to focus on multiple items at a time, a possible reason for reduction in driving activities.

Driving simulation has been employed in a number of studies testing driving-related factors related to the presence of MCI. For example, in one study of a sample of 14 older adults with MCI and 14 age-matched controls aged 65 to 89 [10], those with MCI performed more poorly, but the differences were not statistically significant, potentially due in part to small sample size. Performance on a driving simulator more clearly differentiated those with and without probable Alzheimer’s disease (AD) in a sample of 20 older adults with probable Alzheimer’s disease (a Clinical Dementia Rating of 1) compared to 20 with MCI (a Clinical Dementia Rating of 0.05) [11] and control group participants. Investigators noted only a shorter time to a collision in the MCI group when compared to the unimpaired group [12]. In a more recent driving simulation study of 23 older adults with MCI, 14 with AD, and 38 unimpaired controls, Pavlou and colleagues [13] found that those with MCI drove at a lower speed, left greater space from the vehicle ahead, evidenced problems positioning within a lane, particularly when traffic volume was high, and evidenced impaired reaction time. They concluded that the compensating behaviors used by those with MCI were not sufficient to counterbalance the disease effect. Recent advances in driving simulation testing make it possible to conduct objective tests that are repeatable and may be adjusted to the individual’s capabilities [14] making them a good choice in some instances.

There have been fewer results from naturalistic IVDR (In-Vehicle Data Recorder)-based studies. The subtle changes that occur at the preclinical stage of Alzheimer’s disease (AD) may be detectable through the use of multimodal sensor arrays [15]. For example, data from GPS dataloggers indicated that those with cognitive impairment used fewer distinct routes to reach their usual destination [16]. In a study involving 64 drivers with preclinical AD and 75 unimpaired drivers, jerk (the suddenness or abruptness of the driving), overspeeding, fewer night trips, and reduced distance of trips were noted in those with preclinical AD identified by cerebrospinal fluid biomarkers [17]. Naturalistic driving data has also been used to identify the overall driving style, such as novice, experienced cautious, and experienced reckless [18].

In the LongROAD (Longitudinal Research on Aging Drivers) study in the U.S., recording devices were placed in vehicles of 2977 participants for as long as 45 months. Evidence of either MCI or dementia, found in 64 participants, was ascertained from yearly interviews and medical record review. Analysis suggests that participant age was the strongest predictor of MCI or dementia. Additional predictors were the percentage of trips limited to 15 miles or less from home, race/ethnicity, length of trips, and hard braking occurrences [1]. UDRIVE is a naturalistic study of driver behavior in Europe that will include drivers of cars, trucks, and powered two-wheelers. Driver characteristics, including age and gender, as well as responses to several driver questionnaires, will be related to driver behaviors such as speed, headway, and risky maneuvers [19], but not cognition. Results of on-road driving assessment, neuropsychological tests, and driving surveys in an Australian study included comparisons of safe and unsafe drivers with MCI or no impairment. The investigators reported that drivers with MCI had some additional difficulties at intersections, roundabouts, parking, self-navigation, and straight driving. They did not, however, find that these additional difficulties warranted restrictions on drivers with MCI [1]. In another report from the multi-year, multi-site LongROAD study, investigators found that a substantial proportion of older drivers with poor physical performance reported an increase in strategic self-regulation of their driving behavior [20].

The potential for detecting the subtle early changes in cognitive ability that are reflected in changes in driving behavior has intrigued researchers and clinicians for some time. Comparisons across these different approaches to comparing driving behaviors in those with and without MCI are difficult for several reasons. It is evident that the goals of these studies vary widely. In addition, different driving behaviors are measured in various studies. Overall, however, the use of emerging technologies in naturalistic studies holds promise for developing lower-cost, more widespread detection of the early, subtle changes in Pre-MCI and MCI.

In this ongoing three-year study of older adults’ driving behavior and cognitive status, we compared sensor-derived trip-level driving characteristics between cognitively unimpaired drivers and drivers with pre-MCI or mild cognitive impairment. Each trip-level observation represents a single, aggregated driving episode and includes total distance traveled (km), trip duration (minutes), mean and maximum speed (km/h), mean engine revolutions per minute (RPM), mean throttle position and throttle variability (standard deviation), mean fuel level, and event counts for hard accelerations, hard braking events, and hard turns. All measures reflect raw or aggregated values recorded per trip, with event frequencies expressed as counts and continuous measures summarized at the trip level, without normalization for trip distance or speed.

The purpose of this paper is to report the results of a preliminary analysis of telematic data, including accelerometer data, derived from sensors installed in the vehicles of older drivers who also underwent extensive neuropsychological testing to identify the presence or absence of the subtle cognitive impairment that is characteristic of pre-MCI or Mild Cognitive Impairment (due to the small number of cognitively impaired participants, we combined pre-MCI and MCI into one group for this analysis). Our research question, then, was whether or not we could identify an older adult’s cognitive status (unimpaired or Pre-MCI/MCI) from patterns in their driving behavior.

## 2. Materials and Methods

**Figure 1:**
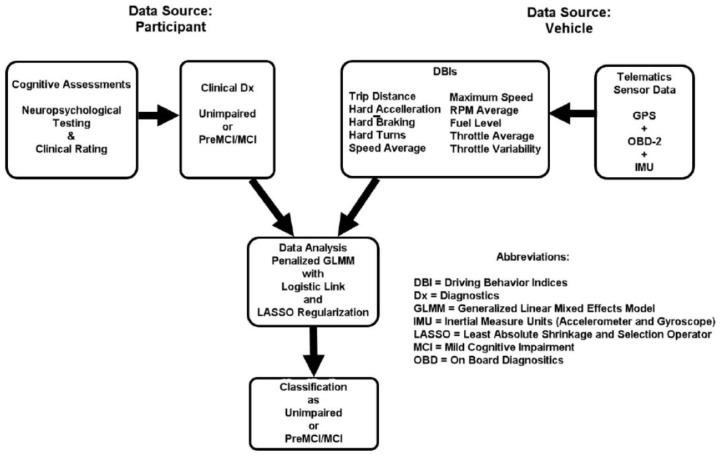
Study Design.

For this exploratory study, we utilized data from an ongoing five-year longitudinal study, focusing specifically on the final quarter of the participant’s first year (Months 9–12) in the study. Three primary data sources were integrated for this analysis: telematic sensor data, demographic information, and cognitive assessment data (see Figure 1).

Telematic Data. Driving behavior was recorded using in-vehicle telematic sensors with sampling intervals ranging from 1 to 5 s. These sensors captured variables such as speed, RPM, throttle position, fuel level, and event-based metrics (e.g., hard braking, acceleration, and hard turns). To standardize the data across varying ranges and reduce data dimensionality, raw sensor data were aggregated at the trip level, producing summary statistics for each trip.

Demographic Survey Data. Demographic information, including age, gender, and years of education, was collected at baseline and updated annually. For this study, the most recent valid demographic data within the study window were used. Any discrepancies were resolved through cross-referencing participant records.

Cognitive Assessment Data. The Montreal Cognitive Assessment (MoCA) [21] was used to screen for general cognitive function and study eligibility, with an education-adjusted cutoff score of 19 or higher to exclude individuals with moderate to severe cognitive impairment. Scores from a battery of neuropsychological and clinical tests administered at the 12-month visit were then used to classify the MoCA-screened participants into two groups: an unimpaired group and a group combining individuals with Pre-MCI and MCI for purposes of the current analysis.

Prior to analysis, all datasets underwent processing. Telematic data were screened for missing timestamps, implausible values (average speeds = 0, for example), and trips shorter than one minute, which were excluded. Demographic and cognitive data were reviewed for completeness and standard formatting. Datasets were then merged using unique participant identifiers and time stamps in a many-to-many merge, linking each trip-level driving record to corresponding demographic and cognitive data.

### 2.1. Sample

This preliminary study included 36 participants, divided into two groups based on cognitive status: unimpaired (n = 23) and Pre-MCI/MCI participants (n = 13). Data were collected from the final three months of the participants’ first year in the study, encompassing a total of 4739 individual trips. Individual driving frequency varied widely, ranging from 30 to 635 trips per participant during the three-month period analyzed. Only the MoCA scores differed significantly between the unimpaired and Pre-MCI/MCI participants (See Table 1).

Eligibility Criteria. Participants were recruited from the community through presentations at senior community centers and health fairs, as well as participant referrals and an organized social media campaign. Eligible participants were aged 65 and older, possessed a vehicle, a valid driver’s license, and car insurance, and had an education-adjusted MoCA score of 19 or higher. Participants were also required to pass vision, hearing, limb strength, and flexibility tests equivalent to those required by state motor vehicle regulations. Potential participants were informed of the study expectations, including installation of telematic sensors and quarterly cognitive testing sessions, at which time telematic data was also downloaded. Enrolled participants received a US $25 gift card at every visit and a US $50 gift card at the final visit.

### 2.2. Cognitive Assessments

#### 2.2.1. Global Cognitive Function

The Montreal Cognitive Assessment (MoCA) was used to screen for global cognitive function and study eligibility. Although only a 10-minute test, it assesses eight cognitive functions, including orientation, short-term and working memory, visuospatial skills, executive function, attention, concentration, and language [21].

#### 2.2.2. Neuropsychological Tests

Executive Function. Divided visual attention, task switching, and cognitive flexibility were assessed using the Trail Making Test (TMT) [22,23,24]. In this task, participants are instructed to connect a series of 25 dots as quickly and accurately as possible. The test consists of two parts: in TMT-A, participants connect numbered dots in ascending order, whereas in TMT-B, they alternate between numbers and letters in sequential order (e.g., 1–A–2–B–3–C, etc.). The primary outcome measure is completion time in seconds. Additionally, a TMT-B/TMT-A ratio was calculated to provide a relatively independent index of executive control [25].

Inhibitory control was assessed using the Stroop Color-Word Test (SCWT) [26]. In this task, participants are instructed to correctly identify the color of the ink in which a word is printed, while avoiding the automatic tendency to read the word itself. This interference between reading and color naming is known as the Stroop effect. All participants were screened for color vision deficiencies prior to testing.

Semantic Memory. In the Loewenstein-Acevedo Scales of Semantic Interference and Learning (LASSI-L), participants learn two 15-word lists drawn from three semantic categories (fruits, musical instruments, and clothing). Words are shown individually on cards and read aloud by the participant. After the initial presentation of List A, participants complete a free-recall trial followed by cued-recall trials for each category. List A is then repeated with another set of cued-recall trials.

Next, List B is presented, followed by a free-recall trial and three cued-recall trials. List B is repeated with additional cued-recall trials. Participants are then asked to recall List A again, completing both free and cued recall trials. After a 20 min delay, participants completed delayed free recall for both lists. Prior research has shown that the LASSI-L is sensitive to distinguishing individuals with MCI from cognitively normal adults [27].

Logical Memory. Logical memory assesses the ability to remember coherent, meaningful information, such as short narratives. Unlike recalling isolated words, it requires retaining connected details of a story. In the Craft Story task, the examiner reads a brief passage aloud, and the participant is asked to recount as many details as possible both immediately and again after a 20 min delay.

Visuospatial and Visuomotor Function. The Benson Figure Drawing (BFD) test is commonly used to evaluate visuospatial cognition in individuals with dementia [28]. During the task, participants are first asked to copy a complex geometric figure, with no time limit imposed, and then to reproduce the figure from memory approximately 20 min later. The copy condition assesses visuospatial and visuomotor constructional abilities, while the delayed recall condition evaluates nonverbal (visual) memory.

Language. Two verbal abilities were evaluated: naming and lexical retrieval. The Multilingual Naming Test (MINT) measures confrontation naming using 32 object pictures, with the score reflecting the number correctly named [29]. It is sensitive to naming difficulties in individuals with MCI or Alzheimer’s disease [30].

Verbal Fluency (VF) assessed lexical retrieval through category fluency (animals) [31] and phonemic fluency (letters P and F) [32]. Scores represent the number of correct words produced. Although performance on fluency tasks relies on executive skills, such as suppressing rule-inconsistent responses, recent work suggests that language processes play the primary role [33].

The Clinical Dementia Rating Scale (CDRS) The Clinical Dementia Rating Scale (CDRS) is a 5-point measure of cognitive impairment severity across six domains: Memory, Orientation, Judgment and Problem-Solving, Community Affairs, Home and Hobbies, and Personal Care [11]. We used the Clinical Dementia Rating-Sum of Boxes (CDR-SB), whose scores range from 0 to 18, as a measure that sums the ratings from the six different cognitive and functional domains, quantifying the severity of dementia. These domains are central to diagnosing and staging Alzheimer’s disease and other dementias in the CDR Sum of Boxes. Ratings are based on interviews with both the participant and an informant (study partner). When discrepancies arose between clinical impressions and test findings, cases were reviewed by a consensus panel of four experts (two neuropsychologists, a neurologist, and a geriatric nursing specialist).

### 2.3. Clinical Diagnosis

A clinical diagnosis of unimpaired, pre-MCI, or MCI was made using an algorithm that has demonstrated reliability and validity in the diagnosis of MCI and dementia [34]. The algorithm combines the Clinical Dementia Rating Scale ratings with the scores in the above-listed neuropsychological tests (see Table 2).

Participants were classified based on this multi-domain algorithm. The Unimpaired group (n = 23) reported no memory problems or functional limitations. They had a CDR-SB score of 0 and showed no signs of cognitive decline during the clinical interview (see Table 2). Their neuropsychological test performance was normal within 1 SD of age and education-adjusted norms across all measures. Those with MCI (n = 3) reported memory complaints and had scores of 1.5 SD or greater, below what is expected using the same normative data on at least one neuropsychological test, and a CDR-SB of 0.5 or above. Individuals with Pre-MCI (n = 10) had either an abnormal CDR with a normal neuropsychological profile or an abnormal score in at least one neuropsychological domain with a normal CDR. For this analysis, participants with MCI and Pre-MCI are combined into a single Pre-MCI/MCI group, to increase statistical power due to the small number of participants in the MCI group. Although Pre-MCI represents the ultra-early stage of cognitive decline (preclinical phase), and MCI denotes a confirmed pathological state, both represent adjacent points on the same continuum of early cognitive decline [35]. Both share the commonality of maintaining independence in daily activities. Additionally, research suggests that pre-MCI and MCI share similar underlying neuropathological changes, such as early Alzheimer’s disease processes [36]. By grouping them, we combined individuals with detectable, yet subtle, cognitive impairment. None of the participants included in this report had dementia.

### 2.4. Telematic Data Collection

An AutoPi (AutoPi.io, Aalborg, Denmark) TMU CM4 built on the Raspberry Pi Compute Model B with a tri-axial accelerometer and GPS was inserted into the vehicle’s OBD (On-Board Diagnostics) port to collect driving behavior data. Raw data was uploaded into a REDCap database for processing and analysis.

### 2.5. Driving Behavior Measures

Measures of driving behavior included overall trip characteristics, vehicle operation, and driving events (see Table 3).

### 2.6. Data Analysis

A penalized generalized linear mixed-effects model (GLMM) with a logistic link and LASSO regularization was used to identify trip-level driving behaviors associated with Pre-MCI/MCI group classification. The binary outcome (0 = cognitively unimpaired; 1 = Pre-MCI/MCI) was modeled as a function of 10 trip-level predictors: distance per trip, number of hard accelerations, number of hard braking events, number of hard turns, mean speed, maximum speed, mean engine RPM, mean fuel level, mean throttle position, and throttle variability. All predictors were standardized (z-scored) so that fixed-effect coefficients represent the change in log-odds of Pre-MCI/MCI group membership associated with a one–standard deviation increase in each driving metric. Standardization was performed using global means and standard deviations prior to model fitting. Given the modest number of participants (N = 36) and the use of driver-level cross-validation with a random-intercept structure, this approach was adopted to promote numerical stability and model convergence. Nonetheless, we acknowledge that future analyses in larger samples should implement a fully leakage-free preprocessing pipeline, in which standardization parameters are estimated within each training fold and applied to held-out drivers.

To account for the hierarchical data structure (thousands of trips nested within 36 drivers), a random intercept for Study_ID was included. Penalization was implemented using glmmLasso with an L1 (LASSO) penalty on the fixed effects. A grid of candidate penalty values (λ = 1, 5, 10, 20, 50) was evaluated using 5-fold cross-validation at the driver level: folds were defined by Study_ID so that all trips from a given driver were held out together. For each λ, models were fit to the training drivers and area under the ROC curve (AUC) was computed on held-out drivers; the value of λ that maximized mean cross-validated AUC (λ = 10) was selected as the optimal penalty.

Following LASSO-based variable selection, all predictors with nonzero penalized coefficients were entered into an unpenalized GLMM (using lme4::glmer) with the same random-effects structure. Following LASSO-based variable selection, predictors with nonzero penalized coefficients were entered into an unpenalized generalized linear mixed-effects model with the same random-effects structure. This post-selection model was used to obtain descriptive coefficient estimates, standard errors, and confidence intervals for interpretability. Because variable selection was data-driven, inferential quantities from the post-selection model should be interpreted cautiously and are considered exploratory rather than confirmatory. Model discrimination was assessed using the AUC and its 95% CI, obtained from ROC analyses performed with pROC.

Given that the effective sample size at the participant level was modest (N = 36 drivers), several steps were taken to reduce overfitting and to obtain a conservative estimate of model performance. First, the use of an L1-penalized GLMM reduced the effective number of parameters by shrinking weak fixed effects toward zero and retaining only the most informative predictors. Second, model tuning was conducted via 5-fold cross-validation at the driver level, such that all trips from a given driver were held out together, providing an estimate of discrimination that reflects generalization to new drivers rather than to additional trips from the same driver. Third, we report both trip-level discrimination (AUC based on all trips) and a more conservative driver-level AUC based on mean predicted probabilities per driver, in order to account for the unequal number of trips contributed by each participant.

RStudio version 2025.09.2 and R version 4.5.2 software was used to conduct the analysis.

### 2.7. Generative AI Use

As part of the manuscript preparation process, Copilot Gen AI (Microsoft 365 Copilot 19.2512.49061.0) was employed to assist with specific technical tasks. The tool was used to facilitate the tabulation of results, including organizing data into structured tables and refining their presentation for clarity and consistency. Additionally, Copilot Gen AI supported the drafting of technical and operational definitions by helping to standardize terminology. All AI-assisted outputs were critically reviewed and edited by the authors to maintain scholarly rigor and accuracy.

## 3. Results

### 3.1. Descriptive Statistics

A total of 4739 trips made by 36 drivers (Study_IDs) were included in the analysis after listwise deletion of missing data. The outcome variable, Overall_Group, was coded 0 for cognitively unimpaired and 1 for Pre-MCI/MC. Across trips, 3440 observations (72.6%) were coded as unimpaired and 1299 (27.4%) as Pre-MCI/MCI.

All driving-behavior predictors were standardized (z-scores) prior to modeling: distance per trip (*dist_per_trip_z*), number of acceleration events (*n_ACC_z*), braking events (*n_BRK_z*), hard turns (*n_HTURN_z*), mean speed (*speed_mean_z*), maximum speed (*speed_max_z*), mean engine RPM (*rpm_mean_z*), mean fuel level (*fuel_mean_z*), mean throttle position (*thro_mean_z*), and variability in throttle (*thro_sd_z*).

### 3.2. Associations Between Trip-Level Driving Behavior Measures

Table 4 presents Pearson correlations among the standardized trip-level driving behavior measures considered as candidate predictors in the mixed-effects models. Correlations were generally small to moderate. The strongest associations were observed between mean speed and maximum speed (*r* = 0.76), between maximum speed and mean RPM (*r* = 0.72), and between mean speed and mean RPM (*r* = −0.63). A moderate negative association was also observed between mean throttle and throttle variability (*r* = −0.41). Although several predictors exhibited shared variance, no correlations exceeded levels typically considered indicative of problematic multicollinearity, supporting the inclusion of these variables in subsequent multivariable modeling.

### 3.3. Penalized Logistic Mixed-Effects Model and Variable Selection

Trip-level telematics data from 36 drivers contributed 4739 valid trips (3440 unimpaired trips and 1299 Pre-MCI/MCI trips). A penalized logistic mixed-effects model with a logit link and a random intercept for driver was used to predict diagnostic group (0 = unimpaired, 1 = Pre-MCI/MCI). Predictors included standardized (z-scored) measures of distance per trip, acceleration events, hard braking events, hard turns, mean speed, maximum speed, mean RPM, mean fuel level, mean throttle, and throttle variability.

To obtain a parsimonious model, an L1 (LASSO) penalty was applied to the fixed effects. Five candidate penalty parameters (λ = 1, 5, 10, 20, 50) were evaluated using 5-fold cross-validation, with all trips from each driver held out together to prevent information leakage. As shown in Table 5, λ = 10 produced the highest mean cross-validated AUC (M = 0.88). With this penalty, the LASSO retained all predictors except maximum speed and hard acceleration, which were shrunk to zero. The trip-level CI may be optimistic; however, the driver-level metrics are the primary outcome.

### 3.4. Unpenalized Logistic Mixed-Effects Model

The predictors retained by the penalized model were then entered into an unpenalized logistic mixed-effects model to obtain unbiased parameter estimates, confidence intervals, and significance tests. The model converged adequately (AIC = 49.90, BIC = 121.00; –2LL = 27.90) and showed a large random-intercept variance (Var = 10,855.00; SD = 34.6), indicating substantial between-driver heterogeneity in baseline odds of Pre-MCI/MCI beyond telematics features. –2LL = 27.90) and showed a large random-intercept variance (Var = 10,855.00; SD = 34.6), indicating substantial between-driver heterogeneity in baseline odds of Pre-MCI/MCI beyond telematics features.

Table 6 summarizes the fixed effects. The model intercept was statistically significant (β = 4.25, SE = 1.94, *z* = 2.19, *p* = 0.029, OR = 70.11). None of the trip-level telematics predictors were significant in the unpenalized model.

All of the predictors were retained by the penalized model except for hard acceleration and maximum speed. The eight retained variables were then entered into an unpenalized logistic mixed-effects model to obtain unbiased parameter estimates, confidence intervals, and significance tests. The model converged adequately (AIC = 49.90, BIC = 121.00; –2LL = 27.90) and showed a large random-intercept variance (Var = 10,855.00; SD = 34.6), indicating substantial between-driver heterogeneity in baseline odds of Pre-MCI/MCI beyond telematics features. Table 5 and Table 6 summarize the fixed effects. The model intercept was statistically significant (β = 4.25, SE = 1.94, *z* = 2.19, *p* = 0.029, OR = 70.11). None of the trip-level telematics predictors were significant in the unpenalized model.

### 3.5. Interpretation of Fixed and Random Effects

Table 7 summarizes the direction and practical interpretation of the fixed effects retained in the post-LASSO logistic mixed-effects model. As noted above, none of the individual predictors reached conventional levels of statistical significance in the unpenalized GLMM, which is expected given the modest number of drivers and the substantial between-driver heterogeneity. Importantly, however, the direction and relative magnitude of effects were consistent with the penalized model and with prior literature on driving behavior in cognitive impairment.

Among the retained predictors, greater throttle variability showed the most consistent direction of association with higher odds of Pre-MCI/MCI, which is consistent with the interpretation that cognitively impaired drivers may exhibit less stable or less consistent pedal control during trips. In contrast, higher mean throttle, higher average speed, and more frequent hard-braking events tended to be observed more often among unimpaired drivers, reflecting comparatively more confident, responsive, or assertive driving behavior

Higher mean engine RPM and shorter or more fragmented trips were directionally associated with increased odds of Pre-MCI/MCI, consistent with less efficient speed regulation and more constrained driving patterns. Although individual predictors should not be interpreted in isolation, the multivariable pattern supported meaningful discrimination between unimpaired and Pre-MCI/MCI drivers, as reflected in the trip-level ROC analysis (AUC = 0.886). These findings indicate that it is the combined configuration of telematics features, rather than any single metric, that most effectively differentiates cognitive status at the trip level.

In the penalized model, the largest-magnitude retained coefficients included throttle variability, mean throttle, mean speed, and hard-braking frequency, suggesting that pedal control and speed-regulation features may be particularly informative when considered jointly within a multivariable framework.

#### Random Effects

The random intercept for Study_ID indicated very large between-driver variability on the log-odds scale (variance = 10,855; SD = 34.6). This reflects substantial heterogeneity in drivers’ baseline probabilities of belonging to the Pre-MCI/MCI group that was not explained by trip-level telematics features alone. In practical terms, trips from the same driver tended to be much more similar to each other than to trips from other drivers, underscoring the importance of accounting for within-driver clustering.

This degree of heterogeneity reinforces the necessity of modeling driver-specific random intercepts to appropriately handle the repeated-measures structure (4739 trips nested within 36 drivers) and to avoid overstating the precision of fixed-effect estimates. Collectively, the random-effects results emphasize that individual differences among drivers play a dominant role in cognitive-status classification, with trip-level behaviors contributing additional, complementary information.

### 3.6. Model Discrimination

The combined telematics profile provided excellent discrimination at the trip level, with a trip-level AUC of 0.886 and a 95% CI.

**Figure 2:**
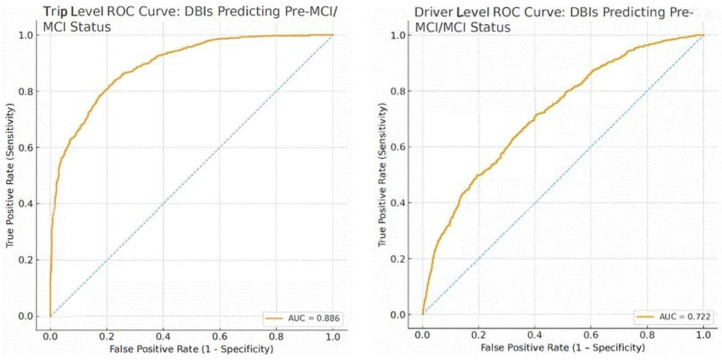
ROC Curve DBI Predicting Pre-MCI/MCI Status Trip and Driver Level.

When predicted probabilities were averaged across trips for each driver (one mean predicted probability per driver), the mixed model demonstrated moderate discrimination, with a driver-level AUC of 0.72, 95% CI [0.682, 0.763] (Table 8 and Figure 2). Driver-level AUC confidence intervals were computed using a nonparametric bootstrap resampling drivers (N = 36), with one mean predicted probability per drive. This more conservative AUC reflects the fact that driver-level ROC curves rely on substantially fewer data points (36 drivers), providing a stricter evaluation of overall classification accuracy.

It is important to note that, although the trip-level model capitalizes on a large number of observations (4739 trips), the underlying number of unique drivers remains small (N = 36). The driver-level AUC of 0.72, therefore, provides a more conservative and appropriate summary of how well the model distinguishes unimpaired from Pre-MCI/MCI drivers, independent of how many trips each driver contributed.

### 3.7. Stability of Predictor Selection Under Grouped Repeated Cross-Validation

To further evaluate the stability of telematics feature selection under correlated predictors and clustered observations, a sensitivity analysis was conducted using repeated grouped cross-validation with LASSO-penalized logistic regression. This analysis assessed how consistently predictors were selected when entire drivers were held out together, preserving the hierarchical structure of trips nested within drivers and preventing information leakage.

Using the trip-level dataset, 100 repetitions of 5-fold cross-validation were performed, with folds defined at the driver level. Within each repetition, model tuning was conducted over a grid of 80 candidate penalty values (λ range: 0.0001–10). Predictor variables were standardized using training-fold means and standard deviations and applied to the corresponding held-out drivers. Model performance was evaluated using a conservative driver-level AUC, computed from mean predicted probabilities aggregated across trips for each driver. The λ value that maximized driver-level AUC was selected within each repetition, and the final model was refit on the full dataset at that λ to identify predictors with nonzero coefficients.

Across repetitions, selected λ values were generally small to moderate (Median λ = 0.094), indicating that modest regularization was sufficient to optimize discrimination. Driver-level AUCs showed moderate predictive performance (Mean AUC = 0.76, ranging from 0.68 to 0.88, consistent with the driver-level discrimination observed in the primary penalized mixed-effects model (see Table 9).

Selection frequencies for individual predictors are presented in Table 10. Mean throttle position was the most stable predictor, selected in 61% of repetitions, followed by hard turns (34%) and throttle variability (34%). Mean speed (23%) and mean RPM (17%) demonstrated moderate selection stability, whereas event-based measures such as hard acceleration, hard braking, and maximum speed were selected less frequently (<12%). Trip distance and fuel level were rarely selected (<10%).

Overall, these results indicate that under repeated grouped resampling, a limited subset of telematics features—particularly those related to throttle control and turning behavior—were consistently retained, while other predictors exhibited greater instability. This pattern is consistent with the presence of moderate collinearity among vehicle operation measures, for which LASSO is known to select one representative predictor from correlated sets. Importantly, the stability analysis reinforces the conclusion that discrimination between unimpaired and Pre-MCI/MCI drivers is driven by the combined multivariate pattern of driving behaviors rather than reliance on any single telematics variable.

## 4. Discussion

Preliminary findings from an exploratory, naturalistic driving study of older drivers are reported. Participants in the study underwent comprehensive cognitive and functional testing quarterly to identify presence of MCI or Pre-MCI vs. remaining unimpaired. At the same time, data was retrieved from telematic sensors, including accelerometers and GPS, installed in their vehicles. The research question addressed is whether or not it is possible to identify patterns of driver behavior in the sensor data related to the subtle cognitive changes in Pre-MCI and MCI.

Results are reported for 36 participants, including 23 who were unimpaired, 10 who had Pre-MCI and 3 with MCI. Employing a penalized generalized linear mixed-effect model (GLMM) with a logistic link and LASSO regularization, patterns of specific behaviors characterizing the MCI group and the unimpaired group were identified. Higher RPM, shorter average trips, and greater throttle variability predicted higher odds of belonging in the MCI group, while more frequent hard braking, hard turns, higher mean speed, and lower mean throttle characterized the unimpaired older driver’s behavior behind the wheel. These results provide preliminary support for the hypothesis that patterns of driver behavior signifying preclinical cognitive impairment can be identified.

The majority of studies directed toward this goal have been based upon driving simulation, which have the advantage of presenting the same stimuli to each participant and also enabling measurement of reaction time. However, the stimuli are not the same as real life with its unpredictable events, and many participants report motion sickness from the use of a driving simulator. Naturalistic studies, on the other hand, can be used to assess driver fitness under real traffic conditions. Both approaches have relatively high cost compared with survey questionnaires [4].

Although this paper presents a preliminary analysis based solely on the final quarter of the first year’s data, the broader project is a five-year longitudinal study. The longitudinal design distinguishes it from prior cross-sectional research and lays the groundwork for more comprehensive and definitive findings in future analyses. Another strength of our study is the use of multiple, well-developed cognitive tests and the combination of clinical ratings (the CDR) with the neuropsychological tests. The algorithm for combining the results has been thoroughly tested [34] and validated in a sample of individuals with MCI against genetic, brain, and plasma biomarkers for Alzheimer’s disease [37], providing a uniform, definitive diagnosis that is difficult to obtain from medical records or the use of a single test meant to be used for screening.

There was some agreement between the results of this study and previously reported studies. We found a pattern of higher RPM, shorter average trips, and greater throttle variability in the MCI group, while more frequent hard braking, hard turns, higher mean speed, and lower average throttle predicted lower odds of MCI. These results are similar to those of Pavlou and colleagues [11], who found that drivers with MCI generally drive at lower speeds. Di and colleagues [1] found in the LongROAD study that hard braking was one of a number of driving behaviors that contribute to prediction of MCI/dementia classification although its reported feature importance ranking was relatively low. More specifically, Babulal and colleagues [38] reported total trips with hard braking at 170.6 for those without evidence of preclinical Alzheimer’s disease on PET scan, but only 104.6 for the group that evidenced preclinical Alzheimer’s disease. Our findings also suggest that hard braking was more typical of the unimpaired drivers, as increased hard braking predicted a lower probability of being classified with Pre-MCI/MCI. Self-regulation may be a significant contributor to changes in driving behavior, but whether or not it is a deliberate response to reduced abilities is unclear and needs further research.

Implications for Future Research. Further research on the mechanisms for changes in driving behavior in Pre-MCI and MCI is needed. It has been suggested that the majority of older drivers with MCI will exercise greater caution while driving. This caution could manifest as slower driving, remaining in the slower lanes, and staying in familiar neighborhoods, resulting in shorter and often less frequent trips. These attempts to compensate for accumulating deficits eventually become insufficient to allow the individual to continue to drive safely. Further research is needed to identify both the volitional and functional capability aspects of these changes in driving behavior with advancing impairment. Larger samples of older drivers are also needed to confirm the patterns that are specific to Pre-MCI and MCI.

Significantly larger samples are needed to confirm the results of the exploratory study reported here. There are also a number of potentially confounding factors that should be considered when larger samples are obtained. Weather conditions, for example, may make driving difficult or even prevent it altogether. A change in physical health, vision, hearing, use of one’s extremities, or a change in medications may also affect driving behavior. Psychosocial and socioeconomic factors and the type of vehicle driven may also play a role.

Limitations. A primary limitation of this study is the relatively small number of drivers (N = 36 including 13 individuals in the combined Pre-MCI/MCI group), despite the large number of trips. As a result, the effective sample size at the participant level is limited, and the precision of individual parameter estimates is constrained. Although the use of LASSO penalization, a random-intercept structure, and driver-level cross-validation reduces the risk of overfitting and partially addresses unequal trip counts, the findings should be viewed as preliminary and hypothesis-generating. In addition, the modest class imbalance (23 unimpaired vs. 13 Pre-MCI/MCI) may influence the stability of the estimated coefficients and AUC. Replication in larger cohorts, with more impaired drivers and more balanced class sizes, is needed to confirm the specific telematics signatures associated with Pre-MCI and MCI.

The paucity of participants with MCI prohibits comparisons between MCI and Pre-MCI or consideration of any differences between those with amnestic MCI and those with non-amnestic MCI. Additionally, the inclusion of the eligibility criterion of a MoCA score ≥ 19 might affect generalizability of the findings to older drivers with more advanced cognitive impairment. Future research, including participants with more advanced cognitive impairment, would also be valuable.

## 5. Conclusions

Although exploratory in nature, the results presented here are promising, suggesting that similar analyses with larger samples may allow confirmation of typical patterns of driving behavior in individuals with Pre-MCI and MCI. In the future, such patterns may become important indicators of changes in cognitive ability that need attention, including further assessment and treatment if indicated.

## Figures and Tables

**Table 1:** Sociodemographic and Sensor Differences Between Unimpaired and MCI Participants.

	Unimpaired (N = 23)		Pre-MCI/MCI (N = 13)		
Descriptive	*M*	*SD*	*M*	*SD*	*p*
Age	75.4	6.3	77.3	6.3	0.398
Years of education	16.2	3.1	16.0	2.8	0.868
MoCA *	26.8	1.8	25.0	2.2	0.010
Average number of Trips	181.1	183.2	162.3	175.8	0.403
	*N*	*%*	*N*	*%*	*p*
Males	14	60.9	9	69.2	0.628
Number of Trips	3440	72.8	1299	27.4	0.770

Notes. MoCA = Montreal Cognitive Assessment Scale [21]. * Significant at *p* < 0.05.

**Table 2:** Algorithm Used in the Clinical Diagnosis.

CDR Sum of Boxes (CDR-SB)	Neuropsychological Test Performance (NP)	
	Normal	Abnormal in at Least One Test
0	Normal	Pre-MCI
0.5–4.0	Pre-MCI	MCI

Note: Normal/Unimpaired: CDR-SB = 0 and Normal NP; Pre-MCI: CDR−SB between 0.5 and 4.0 and NP is normal or CDR−SB = 0 and NP is abnormal; MCI: CDR−SB = 0.5–4.0 and NP is abnormal.

**Table 3:** Telematic-Based Driving Behavior Indices.

Driving Behavior Indices	
Index	Description
Trip Distance	Unit: km Distance of each trip (Seconds)
Number of Hard Accelerations	Unit: No Number of events with Acceleration > 0.3 on the X-axis
Number of Hard Brakings	Unit: No Number of events with Acceleration < −0.3 on the X-axis
Number of Hard Turns	Unit: No Number of events with Acceleration > 0.3 on the Y-axis
Average Speed	Unit: km/h Mean speed in kilometers per hour
Average RPM	Mean revolutions per minute
Fuel Level	Unit: % Percentage of fuel capacity
Throttle Average	Unit: % Percent throttle positioning per trip
Throttle variability	Unit: SD Standard deviation of throttle applications per trip
Maximum Speed	Unit: km/h Highest recorded speed (km/h) across trip

**Table 4:** Correlations Among Trip-Level Driving Behavior Measures.

Variable	1	2	3	4	5	6	7	8	9	10
1. Trip distance	—									
2. Hard accelerations	−0.03	—								
3. Hard braking	−0.06	−0.24	—							
4. Hard turns	−0.02	−0.35	−0.38	—						
5. Mean speed	−0.48	0.10	−0.06	−0.13	—					
6. Maximum speed	0.48	0.29	0.37	0.45	0.76	—				
7. Mean RPM	0.09	−0.09	0.03	0.09	−0.63	0.72	—			
8. Mean fuel level	−0.04	0.05	−0.03	0.06	0.02	−0.01	−0.00	—		
9. Mean throttle	−0.12	−0.18	−0.05	0.11	0.18	0.09	−0.29	0.05	—	
10. Throttle variability	0.15	0.04	−0.01	−0.16	−0.10	0.31	−0.14	−0.03	−0.41	—

**Table 5:** Cross-validated AUC for LASSO penalty parameters (λ) in the penalized logistic mixed-effects model (N = 36 drivers; 4739 trips).

λ	Mean AUC	SD AUC
1	0.77	0.32
5	0.75	0.33
10	0.89	0.33
20	0.82	0.34
50	0.69	0.28

Note. Driver-level 5-fold cross-validation; all trips from each driver remained in the same fold.

**Table 6:** Penalized (LASSO) and unpenalized fixed effects from the logistic mixed-effects model predicting Pre-MCI/MCI (N = 36 drivers; 4739 trips).

	LASSO	Unpenalized				95% CI for B			95% CI for OR	
Predictor (z)	β	β	SE	*z*	*p*	LL	UL	OR	OR LL	OR UL
Intercept	–2.14	4.25	1.94	2.19	0.029	0.44	8.06	70.11	–	–
Distance per trip	0.00	0.28	1.14	0.25	0.806	–1.96	2.52	1.32	0.14	12.37
Fuel level	–0.00	–0.19	1.28	–0.15	0.882	–2.70	2.32	0.83	0.07	10.19
Hard braking	–0.00	–0.72	1.74	–0.41	0.679	–4.13	2.69	0.49	0.08	2.56
Hard turns	–0.00	0.22	2.51	0.09	0.930	–4.70	5.14	1.25	0.01	170.15
RPM (mean)	0.01	0.68	1.46	0.47	0.642	–2.18	3.54	1.97	0.11	34.58
Speed (mean)	0.00	–0.67	1.96	–0.34	0.732	–4.51	3.17	0.51	0.01	23.85
Throttle (mean)	–0.54	–0.64	1.31	–0.49	0.626	–3.21	1.93	0.53	0.04	6.92
Throttle variability)	0.64	0.58	0.12	4.83	0.629	–1.77	2.93	1.79	1.41	2.29

Note. Outcome = 0 (unimpaired) vs. 1 (Pre-MCI/MCI). All predictors standardized (z-scores). βLASSO = penalized coefficients; unpenalized estimates taken from the subsequent GLMM. Hard acceleration and maximum speed were not retained. Together, the strongest retained predictors were throttle variability, mean throttle, hard braking, mean speed, and RPM.

**Table 7:** Interpretation of Fixed Effects from the Post-LASSO Logistic Mixed-Effects Model Predicting Pre-MCI/MCI (N = 36 drivers; 4739 trips).

Predictor	Direction of Association	Practical Interpretation
Intercept	—	Baseline log-odds of Pre-MCI/MCI before considering any trip-level telematics feature.
Distance per trip	↑ distance ↑ odds	Longer trips were associated with slightly higher odds of being in the Pre-MCI/MCI group, but with substantial uncertainty.
Fuel level	↑ fuel level ↓ odds	Higher fuel efficiency or lower fuel consumption showed a small trend toward lower Pre-MCI/MCI risk.
Hard braking	↑ hard braking ↓ odds	More frequent hard-braking events tended to be seen among unimpaired drivers, consistent with more assertive or responsive driving.
Hard turns	↑ hard turns ↑ odds	Hard-turn frequency showed no meaningful pattern differentiating groups.
Mean RPM	↑ RPM ↑ odds	Higher engine workload (higher RPM) trended toward being associated with greater odds of Pre-MCI/MCI.
Mean speed	↑ speed ↓ odds	Faster average driving speed was somewhat more common among unimpaired drivers.
Mean throttle	↑ steady throttle (higher mean) ↓ odds	Drivers with steadier pedal control (higher average throttle) were less likely to be in the Pre-MCI/MCI group, whose drivers tended to have lower average throttle.
Throttle variability	↑ variability ↑ odds	Greater moment-to-moment throttle fluctuation was associated with higher odds of being in the Pre-MCI/MCI group. This was the most consistent behavioral marker across both models.

Note. arrows indicate the direction of the relationship between driving behavior predictors and the odds of having pre-MCI/ MCI.

**Table 8:** ROC-based discrimination of the post-LASSO logistic mixed-effects model predicting Pre-MCI/MCI.

Level of Analysis	AUC	95% CI LL	95% CI UL	Interpretation
Trip-level (4739 trips)	0.886	0.868	0.905	Excellent discrimination
Driver-level (36 drivers, one mean probability per driver)	0.72	0.682	0.763	Moderate discrimination

Note. Driver-level AUC is based on mean predicted probability per driver (N = 36). Lower AUC at the driver level indicates that discrimination accuracy is more conservative when collapsed across trips.

**Table 9:** Distribution of Optimal Penalty Parameters (λ) and Driver-Level AUCs Across Repetitions.

Statistic	λ (Penalty Parameter)	Driver-Level AUC
1st Quartile	0.044	0.68
Median	0.094	0.7
Mean	0.092	0.76
3rd Quartile	0.146	0.74
Maximum	0.226	0.88

**Table 10:** Selection Frequency of Telematics Predictors Across 100 Repeated Grouped Cross-Validation Runs.

Predictor	Selection Frequency (%)
Mean throttle position	61
Hard turns	34
Throttle variability	34
Mean speed	23
Mean RPM	17
Hard acceleration	11
Hard braking	9
Maximum speed	9
Maximum throttle	9
Trip distance	7
Fuel level	6

Note. AUCs were computed at the driver level using one mean predicted probability per driver. λ values correspond to the penalty parameter that maximized driver-level AUC within each repetition.

## Data Availability

The data presented in this study will be available one year after the end of the study.
